# The Role of Artificial Intelligence in Identifying *NF1* Gene Variants and Improving Diagnosis

**DOI:** 10.3390/genes16050560

**Published:** 2025-05-07

**Authors:** Vasiliki Sofia Grech, Kleomenis Lotsaris, Theano Eirini Touma, Vassiliki Kefala, Efstathios Rallis

**Affiliations:** 1 Department of Biomedical Sciences, School of Health and Care Sciences, University of West Attica, GR-12243 Athens, Greece; valiakef@uniwa.gr (V.K.); erallis@uniwa.gr (E.R.); 2 Department of Psychiatry, General Hospital of Athens: “Evaggelismos”, GR-10676 Athens, Greece; psych.kleolots@gmail.com; 3 Child and Adolescent Psychiatrist, General Hospital “Asklepieio Voulas”, GR-16673 Voula, Greece; theanotouma@icloud.com

**Keywords:** *NF1*, gene, children, AI, tumors, VUS, CPT, NGS, phenotype

## Abstract

Neurofibromatosis type 1 (NF1) is an autosomal dominant disorder caused by mutations in the *NF1* gene, typically diagnosed during early childhood and characterized by significant phenotypic heterogeneity. Despite advancements in next-generation sequencing (NGS), the diagnostic process remains challenging due to the gene’s complexity, high mutational burden, and frequent identification of variants of uncertain significance (VUS). This review explores the emerging role of artificial intelligence (AI) in enhancing *NF1* variant detection, classification, and interpretation. A systematic literature search was conducted across PubMed, IEEE Xplore, Google Scholar, and ResearchGate to identify recent studies applying AI technologies to *NF1* genetic analysis, focusing on variant interpretation, structural modeling, tumor classification, and therapeutic prediction. The review highlights the application of AI-based tools such as VEST3, REVEL, ClinPred, and *NF1*-specific models like DITTO and RENOVO-NF1, which have demonstrated improved accuracy in classifying missense variants and reclassifying VUS. Structural modeling platforms like AlphaFold contribute further insights into the impact of *NF1* mutations on neurofibromin structure and function. In addition, deep learning models, such as LTC neural networks, support tumor classification and therapeutic outcome prediction, particularly in *NF1*-associated complications like congenital pseudarthrosis of the tibia (CPT). The integration of AI methodologies offers substantial potential to improve diagnostic precision, enable early intervention, and support personalized medicine approaches. However, key challenges remain, including algorithmic bias, limited data diversity, and the need for functional validation. Ongoing refinement and clinical validation of these tools are essential to ensure their effective implementation and equitable use in NF1 diagnostics.

## 1. Introduction

### NF1: Genetic and Clinical Overview

Neurofibromatosis type 1 (NF1), historically known as von Recklinghausen’s disease, is a genetic disorder that affects multiple systems in the body. It follows an autosomal dominant inheritance pattern and occurs in approximately 1 in every 2500 to 3000 live births. The condition results from inactivating mutations in the *NF1* tumor suppressor gene, located on chromosome 17q11.2, a region known for having one of the highest mutation rates among single-gene disorders in humans [1]. The discovery of the *NF1* gene in 1990 marked a major breakthrough in understanding the molecular mechanisms underlying this disorder [2]. The gene encodes neurofibromin, a large cytoplasmic protein that acts as a GTPase-activating protein, functioning as a negative regulator of the Ras signaling pathway. Under normal circumstances, neurofibromin accelerates the inactivation of Ras, which helps to control cell growth and differentiation. However, when neurofibromin is absent or non-functional due to *NF1* gene mutations, Ras signaling becomes overactive, leading to uncontrolled cell proliferation and tumor formation [3].

NF1 is characterized by a wide range of clinical features, with symptoms typically appearing in early childhood. Among the most common and recognizable signs are dermatological findings such as café-au-lait macules (CALMs), freckling in the axillary or inguinal regions, and Lisch nodules, which are benign hamartomas found in the iris. In addition to these skin manifestations, many children with NF1 experience cognitive and developmental challenges, including learning disabilities, attention-deficit/hyperactivity disorder (ADHD), and features associated with autism spectrum disorders. These neurodevelopmental issues can significantly affect academic performance and social development. Brain imaging often reveals unidentified bright objects (UBOs), particularly in areas such as the basal ganglia and cerebellum, which may correlate with some of the neurological difficulties observed in these patients [4].

Individuals with NF1, both children and adults, also face an increased risk of developing various types of tumors. Among the most common tumors of the central nervous system are optic pathway gliomas, often classified as pilocytic astrocytomas. These typically arise during childhood or adolescence and may impact vision and endocrine function. Other tumor-related complications include the development of neurofibromas, which are benign peripheral nerve sheath tumors that frequently appear around puberty, and congenital plexiform neurofibromas, which may be present earlier and have the potential to grow extensively, sometimes leading to significant morbidity. There is also a risk of malignant transformation of these tumors into malignant peripheral nerve sheath tumors (MPNSTs), which are highly invasive and difficult to treat. Beyond the nervous system, affected individuals may develop pheochromocytomas, gastrointestinal stromal tumors (GISTs), and, in women, an increased risk of breast cancer. Additional complications often include skeletal abnormalities such as scoliosis and tibial dysplasia, as well as cardiovascular issues like renal artery stenosis and congenital heart defects, which are present in approximately 10% of cases [4].

The impact of NF1 on life expectancy is considerable. A population-based study from Finland reported a reduction in lifespan of about 16.5 years for men and 26.1 years for women with NF1, primarily due to the elevated risk of both benign and malignant tumors [5]. These findings emphasize the importance of early diagnosis and timely medical intervention to manage complications and improve patient outcomes.

The diagnosis of NF1 has traditionally been based on clinical criteria established by the National Institutes of Health in 1988. According to these guidelines, the diagnosis requires the presence of at least two of the following features: six or more café-au-lait macules larger than 1.5 cm after puberty or 0.5 cm before puberty, freckling in the underarm or groin regions, two or more neurofibromas or one plexiform neurofibroma, an optic glioma, two or more Lisch nodules, specific bone abnormalities, or a first-degree relative with NF1. However, due to the considerable variability in how the disease presents, even among affected members of the same family, and the potential overlap with other genetic conditions, these diagnostic criteria were updated in 2021 to include molecular genetic testing. The identification of a pathogenic *NF1* variant is now considered an independent diagnostic criterion, a revision particularly beneficial for diagnosing young children who may present with limited clinical features or lack a known family history of the disorder [6].

Integrating genetic testing into the diagnostic process enhances the accuracy of NF1 diagnoses and allows for early detection and individualized management strategies. Recognizing pathogenic *NF1* variants at an early stage enables proactive monitoring and timely intervention, which can help reduce the severity of complications. Furthermore, genetic testing provides valuable information for family planning and helps assess the risk of NF1 in relatives. The integration of molecular diagnostics has thus become a key element in improving both the quality of life and clinical outcomes for individuals affected by NF1.

## 2. Challenges in Genetic Testing and Variant Interpretation

Genetic testing for NF1 presents several key challenges, largely due to the gene’s complexity and the broad spectrum of pathogenic variants observed. The *NF1* gene spans approximately 350 kilobases and comprises 55 constitutive exons and 5 alternatively spliced exons, encoding neurofibromin—a multifunctional protein involved in several critical signaling pathways. This extensive genomic architecture, coupled with high mutational heterogeneity and the absence of recurrent mutation hotspots, complicates the molecular diagnosis of NF1 [7].

One of the primary difficulties in NF1 genetic testing is the gene’s notably high spontaneous mutation rate, reported to be 10 to 100 times greater than that observed in most other human disease-associated genes. This high mutation frequency contributes significantly to the large proportion of sporadic cases, with up to 50% of affected individuals showing no family history, as these mutations often occur de novo [8]. In many sporadic cases, NF1 arises from postzygotic somatic mutations, leading to genetic mosaicism where only a subset of cells carries the *NF1* pathogenic variant. Somatic mosaicism can result in a wide range of clinical presentations, from localized manifestations, such as segmental *NF1*, to generalized but typically milder phenotypes resembling constitutional *NF1.* Detecting mosaic mutations poses considerable challenges, particularly when these variants are present at low variant allele frequencies (VAFs), especially in blood-derived DNA where the proportion of mutant cells may be insufficient for standard diagnostic methods to detect. Consequently, deep sequencing methods or analysis of DNA from other tissues—such as affected skin regions or tumors like neurofibromas—is often necessary to identify low-level mosaic variants. This approach is crucial for patients with atypical, segmentally restricted, or localized NF1 phenotypes where standard blood-based testing may yield false-negative results. It is estimated that approximately 10% of sporadic NF1 cases are caused by postzygotic mutations either absent or present at very low levels in blood lymphocytes, complicating both diagnosis and genetic counseling [9].

Traditional diagnostic approaches using Sanger sequencing, while historically pivotal, are limited by their inability to detect large deletions, duplications, or low-level mosaicism due to their sequential analysis of individual exons. Recent advances have established the combination of next-generation sequencing (NGS) and multiplex ligation-dependent probe amplification (MLPA) as the current gold standard for NF1 molecular diagnostics in clinical practice. NGS allows high-throughput sequencing of all *NF1* coding exons and exon–intron boundaries, efficiently identifying point mutations, small insertions/deletions (indels), and splice site alterations with significantly greater sensitivity than Sanger sequencing. Additionally, NGS read-depth analysis can suggest the presence of copy number variations (CNVs), although larger deletions or duplications may still escape detection by sequencing alone [10]. To address these limitations, MLPA serves as a complementary method for the accurate detection of multi-exon and whole-gene deletions or duplications, which account for a substantial proportion of pathogenic *NF1* variants. The integration of NGS and MLPA thus ensures comprehensive mutational coverage, significantly enhancing diagnostic yield and reliability. This combined strategy is particularly valuable in detecting mosaic mutations, where deep NGS coverage enables the identification of VAFs as low as 7%—a sensitivity level unattainable by Sanger sequencing [10,11]. The practical application and improved efficiency of this approach in clinical settings have been well-recognized, supporting precise genetic confirmation, effective counseling, and appropriate risk assessment for individuals and their families [12].

Yet, there are mosaic mutations with VAFs below 10% that often lead to false-negative results when using standard NGS approaches. This is particularly relevant in cases such as neurodevelopmental disorders (NDDs), where mosaic pathogenic variants may be present at very low levels in peripheral blood and remain undetected by conventional genetic testing. In these situations, high-depth NGS, performed through deep sequencing with a customized mosaicism-specific panel and an optimized bioinformatic pipeline, offers a significant advantage. This method provides ultra-deep coverage, with read depths exceeding 10,000× and reaching up to nearly 70,000× in some samples, allowing for the reliable detection of low-frequency mosaic variants. As seen in the study by Kim et al. [13], this approach enabled the identification of pathogenic *NF1* variants with VAF as low as 2.0% from blood samples of patients with neurocutaneous features associated with NDDs—variants that would likely have been missed by standard NGS or Sanger sequencing. Therefore, high-depth NGS represents a necessary and highly effective diagnostic strategy for detecting low-level somatic mosaicism, which plays a crucial role in the genetic architecture of NDDs [13].

Furthermore, approximately 10% of loss-of-function *NF1* variants affect RNA splicing and are either undetected by conventional DNA-based diagnostic methods or are misinterpreted by in silico splicing prediction tools. This highlights the critical need for RNA-based diagnostic approaches, such as reverse transcription PCR (RT-PCR) and targeted RNA sequencing (RNA-seq), which allow the direct evaluation of transcript-level alterations. Since many splicing abnormalities result from deep intronic variants or cryptic splice site activation—regions typically not covered by standard DNA sequencing—RNA analysis provides essential functional evidence to identify pathogenic variants that might otherwise remain undetected. The ability to directly observe aberrant splicing events at the RNA level enables a more accurate characterization of variant effects, thereby improving the sensitivity and diagnostic precision for NF1 [14].

In a recent study by Koster et al., the diagnostic utility of targeted RNA-seq was demonstrated through the application of a specific enrichment and analysis workflow designed to systematically assess splicing events across the *NF1* transcript. This approach uses either hybridization-based or PCR-based RNA capture methods, followed by sequencing and quantitative assessment of splice junction usage. Central to this workflow is the use of QURNAs (Quantitative Enrichment of Aberrant Splicing Events in Targeted RNAseq), a bioinformatic tool developed to calculate enrichment scores (ERS) for splicing events. This approach is particularly valuable since many splice-disrupting variants in *NF1*, especially those located deep within intronic regions, cannot be reliably predicted by DNA sequence analysis alone [14].

Despite their advantages, RNA-based diagnostic assays present several inherent limitations. A major challenge is the tissue-specific expression of alternative splice isoforms, as *NF1* transcripts display variable splicing patterns depending on the cellular environment. Additionally, RNA is inherently less stable than DNA and more prone to degradation, requiring meticulous handling and processing to preserve transcript integrity for accurate analysis [14].

From a technical perspective, RT-PCR, while commonly used for splice variant validation, has notable limitations. It is labor-intensive, prone to amplification bias, and often constrained by primer design, which may limit analysis to small genomic regions. Its low scalability also makes it less suited for detecting complex or unexpected splicing events, frequently requiring repeated primer redesign. In contrast, targeted RNA sequencing offers broader transcript coverage and quantitative assessment of splicing dynamics, providing a more robust and scalable approach for identifying splice defects [14].

Integrating RNA-seq with DNA variant analysis greatly improves diagnostic accuracy by functionally confirming suspected splice-altering variants and aiding in the reclassification of variants of uncertain significance. RNA analysis also reveals allelic imbalance and transcript destabilization caused by premature termination codons, offering insights into mechanisms like nonsense-mediated mRNA decay. These findings underscore the critical role of RNA-level investigation in identifying pathogenic processes that may be missed by DNA analysis alone [14].

In conclusion, RNA-based diagnostic approaches, particularly targeted RNA-seq, serve as an essential complement to DNA sequencing in the molecular diagnosis of NF1. While both methodological and biological limitations must be considered, the functional data obtained through RNA analysis significantly improve variant interpretation, increase diagnostic yield, and contribute to a deeper understanding of the molecular pathology underlying NF1 [14].

Adding further complexity to NF1 genetic testing is the gene’s extensive allelic heterogeneity, with over 3600 distinct pathogenic variants reported. These so called variants of uncertain significance (VUS), are not confined to coding regions but can also occur deep within intronic sequences, affecting splicing and making detection more challenging [7]. Approximately 46% of NF1 patients carry extremely rare or private mutations—unique to individuals or families—complicating variant interpretation, especially for missense or in-frame indel variants where clinical impact remains uncertain [15]. Another significant factor is the presence of CNVs, including single-exon deletions and whole-gene deletions, which account for approximately 5–11% of NF1 cases. These “*NF1* microdeletions” are often associated with more severe phenotypes, known as *NF1* microdeletion syndrome [16]. Therefore, a comprehensive genetic testing protocol, including both DNA- and RNA-based methods such as cDNA sequencing, analysis of exon–intron boundaries, and CNV assessment, is necessary to maximize diagnostic yield. Despite such approaches, the interpretation of VUS remains a persistent hurdle, limiting the detection rate for clearly pathogenic variants in some cases [17].

It is now well-established that diagnosing NF1 can be challenging, particularly in children and young adults who may present only with dermatological signs and lack other characteristic features or a known family history. In such cases, relying solely on clinical criteria may be insufficient, especially for individuals with milder phenotypes that do not meet the classical diagnostic thresholds. Certain pathogenic *NF1* variants, such as p.Met992del and those affecting Arg1809 or Met1149, are known to cause these atypical or mild presentations, where symptoms may appear later in life. Therefore, genetic testing plays a critical role in confirming the diagnosis, even when clinical signs are limited [17,18].

However, a negative genetic test result reduces—but does not completely rule out—the possibility of constitutional NF1, highlighting the need to continuously improve the sensitivity and accuracy of molecular diagnostics. In this context, emerging technologies, including artificial intelligence (AI)-based tools, are already contributing to enhancing variant interpretation, improving the detection of mosaicism, and supporting clinical decision-making.

Despite the diagnostic value of genetic testing, testing is sometimes deferred, particularly in mild or non-specific cases, with a watchful waiting approach often being adopted until further clinical features develop. This reflects broader concerns, including financial, ethical, and psychological considerations [17]. Nevertheless, with the advancement of AI-driven approaches, there is growing potential to better guide such decisions, offering predictive insights and personalized recommendations that may help clinicians and patients navigate these complex choices.

## 3. AI in *NF1* Variants

### 3.1. Variant Interpretation Tools

The increasing use of high-efficiency sequencing technologies such as NGS has significantly enhanced the detection of genetic variants in disease-causing genes (DCGs), including the *NF1* gene. This advancement, driven largely by progress in bioinformatics, improves not only our understanding of the genetic basis of complex disorders, but also paves the way for more accurate diagnoses, earlier interventions, and targeted therapeutic strategies.

The interpretation of genetic variants remains a major challenge in clinical genomics, particularly given the high volume of rare or novel variants uncovered by NGS. Bioinformatic and deep learning tools have become essential for prioritizing these variants and assessing their potential pathogenicity, especially in the context of diseases where experimental validation is often unfeasible. A central strategy in variant interpretation involves integrating multiple layers of computational predictions, including variant effect on protein function, RNA splicing, evolutionary conservation, and genome-wide functional potential. The combination of these complementary approaches increases the reliability of pathogenicity assessments and helps overcome the inherent limitations of any single predictive method. At the core of coding variant interpretation are functional effect predictors designed to assess the pathogenicity of missense mutations. Among these, REVEL (Rare Exome Variant Ensemble Learner) and EVE (evolutionary model of variant effect) represent two of the most advanced approaches. REVEL employs an ensemble machine learning framework that integrates outputs from individual predictors such as SIFT, PolyPhen-2, MutationAssessor, and GERP, achieving superior accuracy in classifying rare missense variants with low allele frequencies [19]. In contrast, EVE introduces an unsupervised deep generative modeling strategy that leverages evolutionary sequence data across thousands of species. By modeling the distribution of naturally occurring protein sequence variation, EVE quantifies evolutionary constraints without the need for clinical labels, producing predictions that perform on par with high-throughput functional assays [20]. Together, REVEL and EVE exemplify two complementary philosophies in variant effect prediction—one based on ensemble supervised learning, and the other on fundamental evolutionary modeling.

In addition to coding sequence changes, splicing disruption is a well established mechanism of genetic disease, making the accurate prediction of splice-altering variants essential for comprehensive genetic diagnostics. SpliceAI, a deep learning-based model, offers state-of-the-art performance by assessing both proximal and distal effects on donor and acceptor splice sites across broad genomic contexts. Unlike traditional computational tools which rely on motif-based or statistical models and are limited to canonical splice regions, SpliceAI provides a transcript-wide view, enabling the detection of both canonical and non-canonical splice-altering variants [21]. In a study evaluating 285 experimentally confirmed *NF1* variants with experimentally validated splicing outcomes, SpliceAI achieved a sensitivity of 94.5%, specificity of 94.3%, and an AUC of 0.975, highlighting its accuracy and clinical utility. Its superior performance effectively identifies a broader range of splice-altering variants, including those beyond typical splice site boundaries. This makes SpliceAI a powerful in silico tool for a transcript-wide assessment of potential splicing alterations, significantly improving the identification of non-obvious pathogenic variants improving the interpretation of *NF1* variants and reducing reliance on labor-intensive RNA analyses in clinical diagnostics [22].

Another important dimension in variant interpretation is the evolutionary conservation of genomic positions, as regions under strong purifying selection are more likely to harbor functionally important elements. The comparative statistical Genomic Evolutionary Rate Profiling (GERP) score quantifies this conservation by measuring the reduction in observed substitutions relative to neutral expectations across multiple species. While GERP effectively identifies long-term constrained sites, recent population genetic models highlight its reduced power in detecting functional elements that experience rapid evolutionary turnover, particularly within non-coding regions [23]. This limitation emphasizes the need to integrate conservation data with additional functional annotations.

To extend variant interpretation beyond protein-coding regions, genome-wide functional annotation tools such as GenoCanyon provide valuable insights. GenoCanyon applies a subset of AI, called unsupervised statistical learning, to integrate diverse genomic annotations to estimate the functional potential of each nucleotide across the genome [24]. Importantly, it enables the prediction of functional non-coding regions, which are increasingly recognized as significant contributors to disease.

Although numerous tools have been developed for variant interpretation, representative examples such as REVEL and EVE for coding variant effect prediction, SpliceAI for splicing prediction, GERP for conservation analysis, and GenoCanyon for genome-wide functional annotation illustrate how integrating diverse strategies can enhance diagnostic yield and improve variant prioritization in genetic diagnostics. The integration of these complementary tools not only enhances diagnostic yield but also provides a scalable approach to variant interpretation in the era of genomic medicine.

Building on these developments, AI contributes by effectively processing and interpreting the large volumes of data generated, supporting more precise variant analysis, pattern recognition, and informed clinical decision-making.

The interpretation of these variants, particularly missense changes, remains a major clinical challenge in NF1. Nearly half of the *NF1* variants listed in ClinVar, a central repository for clinically annotated variants, are classified as VUS, with missense mutations constituting the vast majority [25]. This ambiguity is especially problematic in *NF1*, where diagnosis is often suspected early in life, yet canonical features may not be fully present and de novo mutations are frequent [26,27].

To address this challenge, recent efforts have turned to AI and machine learning-based computational predictor, the so-called “metapredictors”, to support the pathogenicity assessment of missense variants. These algorithms are typically trained on large, curated datasets and incorporate a variety of features including protein structure, evolutionary conservation, and biochemical properties [28]. In this context, a recent study by Accetturo et al. [29] assessed the predictive performance of three AI-driven tools—VEST3, REVEL, and ClinPred—specifically applied to the interpretation of *NF1* missense variants extracted from ClinVar [29].

These tools, although developed independently and based on different underlying models, were evaluated for their ability to reclassify VUS into two main categories: either as likely benign or likely pathogenic. Each predictor employs a machine learning-based scoring system but differs in methodology and feature integration: VEST3 (Variant Effect Scoring Tool, version 3) operates by combining various types of data, including evolutionary conservation, protein sequence features, and structural properties, using supervised machine learning models trained on well-characterized pathogenic and benign variants from the Human Gene Mutation Database (HGMD). This allows VEST3 to evaluate how likely a given amino acid substitution is to disrupt protein function based on the biological context of the residue [30]. REVEL, as already mentioned, follows a similar ensemble approach but distinguishes itself by explicitly integrating the output of multiple individual prediction tools, focusing on maximizing accuracy for rare missense changes [19].

ClinPred also employs machine learning but was trained directly on ClinVar data, using a set of variants with high-confidence clinical classifications. It combines evolutionary conservation, protein functional annotations, and outputs from other tools, along with clinical evidence, to predict variant pathogenicity [28]. While all three predictors use overlapping types of information, their training datasets, feature selection, and algorithmic approaches differ, leading to variability in their outputs. This ensemble methodology aims to improve prediction accuracy by leveraging diverse sources of variant-related information, though as the study shows, gene-specific fine-tuning remains necessary for optimal performance.

It is worth noting that when the numerical scores produced by these tools for the same mutations were compared, the correlation between them was relatively weak. This is likely because each tool focuses on different features of the mutations, such as evolutionary conservation, protein structure, or physicochemical properties. However, by combining the specific scoring thresholds from these tools, the authors were able to reduce the proportion of VUS from 88% to approximately 52%. This approach ultimately improved the distinction between likely benign and likely pathogenic variants, with minimal misclassification, particularly in protein domains associated with clinical phenotypes [29].

Despite these promising results, several limitations were noted. First, while the risk of training set circularity (i.e., overlap between training and testing data) was carefully evaluated and found to be minimal, it cannot be entirely excluded. Nevertheless, the datasets used in the training of VEST3, REVEL, and ClinPred were largely distinct from the *NF1*-specific ClinVar entries evaluated in this study. Such separation minimizes the risk of data circularity and strengthens the validity of the performance assessment conducted in this work. Additionally, while these tools provide valuable probabilistic estimates, they do not replace clinical judgment or the need for experimental validation [29].

These results contribute to a growing body of evidence supporting the use of customized computational models to enhance the clinical utility of genetic data, particularly in complex, multisystem disorders such as NF1.

### 3.2. Predicting Pathogenicity of NF1 Variants

Predicting the pathogenicity of *NF1* variants is essential for identifying clinically relevant mutations and guiding precision medicine efforts. In this context, a recent study by Chen et al. [31] applied a comprehensive AI-driven computational framework to predict the pathogenicity of missense mutations in the *NF1* gene, with a specific focus on cysteine substitutions. These residues play a vital role in protein folding, disulfide bond formation, and maintaining the structural stability of neurofibromin. Thus, mutations affecting cysteine sites can disrupt protein function and are likely to contribute to the molecular mechanisms underlying NF1 [31,32].

The study began by collecting and curating mutation data from major genomic databases, including UniProt, the Human Gene Mutation Database (HGMD), and ClinVar. To ensure clinical relevance, the authors applied stringent filters based on the American College of Medical Genetics and Genomics (ACMG) guidelines [33], retaining only variants classified as pathogenic or likely pathogenic. This process retained only those variants classified as pathogenic or likely pathogenic and resulted in a curated set of 204 non-synonymous variants, with a particular focus on cysteine-related mutations. This high-confidence dataset served as the foundation for the study’s comprehensive in silico pathogenicity assessment [31].

To predict the functional impact of these variants, the study utilized PredictSNP2, an ensemble classifier that integrates several established algorithms including SIFT, PolyPhen-1 and -2, SNAP, PANTHER, PhD-SNP, MAPP, and MAGPIE. PredictSNP2 outputs a normalized score between 0 and 1, with higher scores indicating greater probability of pathogenicity. By leveraging the strengths of multiple predictive models, this approach increases the robustness and accuracy of the pathogenicity predictions. The scoring system takes into account evolutionary conservation, biochemical properties, and structural features of the amino acid changes, providing a comprehensive evaluation of each variant [31].

In addition to pathogenicity scoring, the study explored the biophysical consequences of the mutations using the iStable platform, which integrates iMutant 2.0 and MUpro. Both tools are based on machine learning techniques, including support vector machines (SVMs) and neural networks, to estimate the change in protein stability (ΔΔG) caused by the mutations. These predictions provided critical insights into how amino acid substitutions might destabilize the neurofibromin protein, with lower stability often correlating with impaired protein function. This layer of analysis is essential, as even mutations that do not completely eliminate protein production can cause pathogenic effects by reducing structural integrity or altering functional domains [31].

Evolutionary conservation analysis was also performed using ConSurf, which applies a Bayesian algorithm to multiple sequence alignments to determine conservation scores for each residue [34]. The evolutionary significance was further analyzed using Align-GVGD, an AI tool that combines scores to assess how different the substituted amino acids are from the original residues across evolutionary time. This analysis reinforces the pathogenicity predictions by highlighting mutations that occur at evolutionarily conserved sites where functional tolerance to variation is low [31].

To evaluate the structural consequences of the predicted pathogenic mutations, the study employed AlphaFold3, a deep learning-based tool for high-accuracy three-dimensional protein structure prediction. By modeling both the wild-type and mutant neurofibromin structures, the researchers were able to visualize how specific mutations altered protein folding and chemical bonding [35]. Additionally, the HOPE bioinformatic server was used to complement these findings by providing physicochemical annotations of the mutations, such as changes in residue size, charge, and hydrophobicity, further enhancing the understanding of their potential impact on protein stability [31,36].

The study also incorporated the SNPeffect database to predict the potential phenotypic consequences of the mutations. SNPeffect integrates a suite of traditional computational biology tools, including TANGO (aggregation propensity), WALTZ (amyloidogenic potential), LIMBO (chaperone-binding likelihood), and FoldX (protein stability effects), offering a multi-dimensional analysis of how each variant might influence protein behavior at the cellular level. This additional layer of analysis is particularly valuable for identifying mutations that may contribute to disease through mechanisms like protein misfolding or aggregation, which are common features in genetic disorders [31].

Through this comprehensive computational approach, Chen et al. [31] successfully prioritized potentially pathogenic *NF1* mutations, particularly those affecting cysteine residues, without the immediate need for costly and time-consuming laboratory experiments. Notably, the study identified three variants—C379R, R1000C, and C1016Y—as consistently exhibiting high pathogenicity scores and reduced protein stability. Among these, R1000C was distinguished by a marked increase in aggregation propensity, a characteristic associated with protein misfolding disorders. In contrast, C379R and C1016Y did not significantly affect aggregation potential, and none of the three mutations were found to alter amyloid-forming capacity or chaperone-binding behavior [31].

These findings highlight the utility of AI-driven analyses in enhancing the molecular understanding of *NF1* mutations and highlight potential avenues for therapeutic intervention. The detailed characterization of cysteine mutations opens the door for the development of mutation-specific treatments, such as small molecules that restore protein stability or gene therapy approaches designed to correct the underlying genetic defects. These strategies exemplify the potential of precision medicine to tailor treatments according to individual genetic profiles, moving beyond symptomatic management toward targeting the molecular basis of the disease [31].

Despite the strengths of this AI-powered approach, the authors acknowledge important limitations. The study relies exclusively on computational predictions, which, while powerful, require experimental validation to confirm their biological relevance. Furthermore, the clinical significance of these variants across diverse patient populations remains to be fully explored. Future research should aim to bridge the gap between computational findings and clinical outcomes, incorporating experimental assays and patient-derived data to better understand how specific mutations influence the wide phenotypic spectrum observed in *NF1*. Additionally, the study did not fully account for population-specific genetic variation, which may affect the generalizability of the results. Expanding future analyses to include genetically diverse cohorts will be essential for improving the clinical relevance of pathogenicity predictions [31].

In conclusion, this study demonstrates the power of AI-based computational tools in predicting the pathogenicity of *NF1* mutations and provides a valuable framework for guiding experimental studies and therapeutic development. This integrative approach represents an important step toward precision medicine for NF1, offering new insights into mutation prioritization, functional assessment, and targeted intervention strategies.

Another AI tool that stands out in the prediction of *NF1* variant pathogenicity is DITTO (Deep Integration for Transcriptomic and Translational Omics), developed by Mamidi et al. [37]. What differentiates DITTO from other models is its unique ability to consider the different conformational states of proteins when evaluating the functional impact of genetic variants. Rather than relying solely on static structural models, DITTO integrates dynamic structural information, capturing how mutations may affect a protein’s behavior across its various functional forms, such as open and closed conformations. This is particularly relevant for complex proteins like neurofibromin, where structural flexibility plays a key role in protein function and regulation [37].

It was trained on a large-scale dataset of over 696,000 variants from ClinVar, each annotated with functional and frequency data through OpenCravat [38]. The architecture of DITTO integrates multi-level biological information, including genomic sequence context, transcriptomic characteristics, and proteomic structure predictions, to classify variants as either pathogenic or benign. In addition, DITTO incorporates other tools like machine learning SAAFEC-seq and statistic bioinformatics Site Directed Mutator to assess protein stability changes (ΔΔG), alongside AlphaFold-based modeling to evaluate the structural effects of mutations on neurofibromin [37].

When applied to the *NF1* variant dataset from the Leiden Open Variation Database (LOVD), the model accurately classified 877 out of 901 variants (98%) as either pathogenic or benign, performing an exceptionally high classification accuracy. Importantly, beyond binary classification, DITTO enables mechanistic insights into the functional consequences of specific mutations by analyzing their effects on protein stability in different conformational states. The identified *NF1* variant p.G848R, involving the substitution of glycine with arginine at position 848, was predicted to be deleterious due to its destabilizing effect on the protein. The model revealed that while the mutation caused only a mild reduction in stability in the closed conformation of neurofibromin, it led to a significantly greater loss of stability in the open conformation, which is crucial for certain aspects of the protein’s activity [37].

This example illustrates how the dynamic nature of protein structures can significantly influence the pathogenic potential of genetic variants—a factor often missed by traditional static models. By capturing these context-dependent effects, DITTO adds an important layer of biological interpretation, particularly for variants located in regulatory or flexible regions of *NF1*. Furthermore, by evaluating how mutations impact the transition between different structural states, the model contributes valuable mechanistic insights into genotype–phenotype relationships, which are especially relevant in disorders like NF1 that exhibit variable expressivity [37].

Despite its strengths, DITTO has certain limitations. Like all computational tools, its predictive performance relies on the quality and completeness of the input data. Although trained on a large and diverse dataset, potential biases may still exist, particularly for variant types or ethnic populations that are underrepresented. Additionally, while DITTO provides important functional predictions, experimental validation remains essential to confirm these computational findings and to inform clinical decision-making. The model’s applicability may also be limited for variants located outside well-characterized regions where structural or functional annotations are lacking [37].

## 4. Clinical Pipelines

### 4.1. Tumor Detection

NF1 is commonly characterized by the formation of tumors, primarily affecting the nervous system, especially the peripheral nerves. However, due to the wide variability in clinical presentation and the progressive nature of many tumors, achieving an early and accurate diagnosis remains a major challenge. Acknowledging this issue, in 2023, Bidollahkhany et al. [39] developed a deep learning model, GENIE-NF-AI, aimed at distinguishing tumors associated with NF1 by using gene expression data from the AACR GENIE (American Association for Cancer Research Genomics Evidence Neoplasia Information Exchange) project [40]. Their study included 71,572 tumor samples, each described by 973 gene expression features that indicate the activity levels of specific genes. These gene expression profiles provide valuable insights into whether a tumor may be linked to NF1, even in cases where clinical symptoms have not yet fully developed [39].

To construct this predictive model, the researchers employed a liquid neural network (LTC) architecture, incorporating two Long Short-Term Memory (LSTM) layers—deep learning components particularly well-suited for processing sequential biological data such as gene expression patterns. The model was trained using standard deep learning techniques, including the use of dropout layers to reduce the risk of overfitting and learning rate scheduling to optimize model performance. Additionally, clinical variables such as patient age and sex were included in the analysis to control for potential confounding factors, thereby enhancing the model’s robustness and generalizability [39].

The performance of the model was rigorously evaluated using several key metrics, including accuracy, precision, recall, and F1-score. The model achieved a remarkable test accuracy of 99.86%, with perfect precision and recall values of 1.00 for identifying NF1-related tumors. This indicates that the model successfully detected all NF1-associated tumors without incorrectly classifying any non-NF1 tumors as NF1-positive. Compared to previous machine learning approaches and traditional clinical diagnostic methods, this deep learning model demonstrated statistically significant improvements in classification performance (*p* < 0.001) [39].

Beyond achieving high predictive accuracy, the researchers prioritized the interpretability of their model, recognizing that this is crucial for clinical application where understanding the basis of predictions is essential. Since the core deep learning approach functions as a black-box model, they employed explainable AI techniques to enhance transparency. Specifically, they used glass-box models (such as logistic regression) and added explainable layers on top of the black-box model to better interpret the model’s decisions. Through this approach, they were able to analyze the contribution of individual gene expression features to the model’s predictions. This interpretability analysis confirmed that the model’s outputs were driven by meaningful biological signals rather than random patterns in the data, significantly improving its potential utility and trustworthiness in clinical settings [39].

Despite these promising results, the study acknowledges several important limitations. First, while the model achieved excellent performance in detecting NF1-related tumors, its ability to correctly identify non-NF1 tumors was lower, reflecting reduced recall for this group. This limitation could restrict the model’s application in broader tumor classification scenarios. Second, although the dataset was large, it was based on pre-labeled retrospective data, which may introduce bias and limit the model’s generalizability to real-world clinical environments where data can be incomplete or more heterogeneous. Additionally, the binary classification approach (NF1 vs. non-NF1) does not capture the full diversity of NF1 tumor subtypes, nor does it consider patients with borderline or overlapping genetic syndromes. Finally, while the model focused exclusively on gene expression data, the inclusion of other biological data types—such as proteomic, epigenomic, or imaging data—could further enhance its predictive power and relevance for clinical decision-making in the future [39].

This year, Bonetti et al. [41] introduced RENOVO-NF1, an AI-powered tool specifically developed to interpret *NF1* missense variants. Adapted from the earlier RENOVO algorithm [42], a random forest-based model for general variant interpretation, RENOVO-NF1, was retrained using *NF1*-specific datasets to improve accuracy for this gene. A key feature of the model is the Pathogenicity Likelihood Score (PLS), which reflects the proportion of decision trees that classify a variant as pathogenic. To evaluate performance, the authors applied a “database archaeology” approach, distinguishing between variants consistently classified over time (“stable”) and those initially labeled as uncertain but later reclassified (“unstable”). This enabled both retrospective and prospective validation. RENOVO-NF1 achieved 98.6% accuracy during training and maintained strong performance on new data, with 96.5% accuracy on a 2020 test set of stable variants [41].

Even on a more challenging 2024 set—containing variants that were uncertain in 2020 but later reclassified—the model maintained a respectable 82% accuracy. RENOVO-NF1 performed particularly well on non-synonymous single nucleotide variants (SNVs), which result in amino acid changes in the neurofibromin protein, achieving over 96% accuracy for this variant type. Notably, the model enabled the reclassification of 4412 *NF1* missense variants previously labeled as variants of uncertain significance (VUS), with 79% confidently categorized as likely benign or likely pathogenic. This has direct clinical implications, supporting earlier diagnosis, tumor surveillance, and management of NF1-associated conditions such as plexiform neurofibromas and malignant peripheral nerve sheath tumors [41,43].

Despite the promising performance of RENOVO-NF1, the study does present certain limitations. While the model was highly accurate for missense variants, it showed reduced accuracy for non-missense changes, particularly intronic variants, where the absence of key predictive features such as protein-level impact impairs model reliability. This limitation reflects the model’s dependence on well-characterized variant types and highlights the ongoing need for complementary methods—such as functional assays—for non-coding or splicing-region variants [41].

Furthermore, although RENOVO-NF1 significantly accelerates variant interpretation, it does not replace the ACMG guidelines, which remain the gold standard for clinical classification. Instead, the model serves as a prioritization tool—identifying likely pathogenic variants that should undergo full ACMG validation. This is especially valuable in clinical contexts lacking family history or segregation data, such as cases with de novo *NF1* variants, where early identification is otherwise delayed [41].

Finally, while RENOVO-NF1′s training and validation on retrospective ClinVar data ensures robustness, its generalizability to real-time clinical variant discovery depends on continuous updates to both data and model parameters. The slow rate of variant reclassification in public databases—roughly one reclassified variant per 30 new VUS—suggests that predictive tools like RENOVO play a critical role in bridging this gap until more definitive evidence becomes available [41].

### 4.2. Therapeutic Prediction

It is well-established that NF1 presents with a wide spectrum of clinical features affecting multiple organ systems. Among these, one of the most severe skeletal complications is the congenital pseudarthrosis of the tibia (CPT), a rare, debilitating condition characterized by spontaneous fractures that fail to heal, typically manifesting in early childhood [44].

Clinical data show that between 50% and 90% of CPT cases are associated with *NF1* variants, featuring a strong genetic link between the two conditions. Research suggests that up to 80% of individuals with CPT carry *NF1* mutations, and that the complete loss of *NF1* function, through a “double-hit” mechanism involving both *NF1*-haploinsufficient and *NF1*-null cells, may be necessary for the disease to develop. These mutations disrupt the RAS/MAPK signaling pathway, which plays a crucial role in bone remodeling. As a result, individuals with NF1 and CPT often exhibit abnormal osteoclast differentiation, leading to excessive bone resorption and impaired osteoblast function, which reduces bone formation. This dual defect significantly hinders fracture healing and often limits the effectiveness of standard orthopedic treatments. Many patients undergo multiple surgeries, and in severe cases, amputation may be the only viable option [44].

Nowadays, treatment approaches typically involve the surgical removal of pathological tissue combined with the application of bone morphogenetic proteins (BMPs). However, the outcomes of these therapies remain inconsistent, particularly in pediatric patients [44]. The situation is further complicated by the heterogeneity of *NF1*-related disease mechanisms and the rarity of CPT, both of which pose significant challenges to conducting large-scale clinical trials [45,46].

In response to these challenges, Carlier et al. [46] developed a virtual clinical trial model involving 200 virtual patients, where each simulated subject received either no treatment or BMP therapy. This in silico framework was based on a previously validated multiscale mechanistic model of murine bone regeneration and incorporated key biological processes relevant to CPT. The model accounted for eight critical parameters reflecting *NF1*-associated cellular dysfunction, including enhanced fibrous tissue proliferation, impaired cartilage and bone formation, and disrupted osteogenic and angiogenic signaling. Machine learning played a pivotal role in this approach by enabling the simulation of therapeutic outcomes and facilitating the analysis of complex, nonlinear interactions among these parameters [46].

Healing outcomes were modeled both with and without BMP therapy, allowing AI algorithms to stratify subjects based on their predicted treatment response. The analysis revealed significant variability in outcomes where BMP therapy produced notable improvement in some virtual patients (responders), showed no benefit in others (non-responders), and even led to negative effects in a small subset (adverse responders). Additionally, the study identified a group of asymptomatic virtual patients whose condition remained largely unaffected regardless of treatment [46].

Crucially, the model also facilitated the identification of predictive biomarkers for treatment response. Parameters such as the rate of cartilage formation (Pmc), osteogenic differentiation (Y11), and endochondral ossification (Y3cb) emerged as strong predictors of BMP responsiveness. These biological markers are closely linked to the *NF1* mutation status of cells, emphasizing the gene’s pivotal role in influencing CPT severity and treatment outcomes, and highlighting the complex genotype–phenotype relationships involved in NF1-associated CPT. The model’s predictive accuracy was further validated through correlation analyses and receiver operating characteristic (ROC) curves, supporting its potential for biomarker discovery and the development of personalized therapies [46].

Importantly, this in silico trial approach offers an ethically sound pathway for exploring therapeutic strategies in vulnerable populations, such as children with rare bone disorders, where conventional clinical trials may face ethical or practical barriers. By simulating treatment responses virtually, researchers can evaluate potential risks and benefits before proceeding with real-world interventions, positioning this model as a valuable tool for preclinical decision-making.

Despite its innovation, the study presents certain limitations. The in silico trial was based on murine biology and modeled only 200 virtual subjects, limiting its capacity to fully capture the complexity of human CPT. Furthermore, the model did not include mechanoregulatory influences or the effects of different surgical techniques, BMP dosages, and prior treatments—all of which could influence clinical outcomes. The subgroup of adverse responders was particularly small, preventing strong statistical conclusions for this cohort. Additionally, the model used a uniform sampling of the *NF1* parameter space, which may not fully reflect the real distribution of cellular behaviors in patients [46].

In a similar context, a more recent study by Xu et al. [47] used human genetic data to investigate a rare case of CPT within a five-generation Chinese family. The researchers identified a novel truncating mutation in the *NF1* gene (c.871G>T; p.E291*), with affected individuals, such as the proband in this study, typically presenting early in life with severe bone deformities, recurrent fractures, and impaired bone healing. To explore the genetic basis and functional consequences of this mutation, the researchers employed a range of bioinformatics tools at various stages of their investigation [47].

Advanced computational approaches played a central role in the analysis of whole-exome sequencing (WES) data, followed by Sanger sequencing to confirm the presence of the identified mutation. Bioinformatic algorithms enabled efficient filtering, alignment, and detection of the pathogenic variant, demonstrating the diagnostic power of WES and paving the way for potential gene therapy strategies. To investigate the functional impact of the mutation, structural modeling tools were used to generate a three-dimensional representation of the neurofibromin protein through platforms such as PyMOL. This analysis revealed that the p.E291* variant leads to the loss of several critical protein domains, including the cysteine-serine-rich domain (CSRD), GAP-related domain (GRD), Sec14-homologous and pleckstrin homology domain (SEC14-PH), and the C-terminal domain (CTD)—all of which are essential for the proper regulation of Ras/MAPK signaling [47].

Furthermore, evolutionary conservation analysis using platforms like the ConSurf server demonstrated that the glutamic acid residue at position 291 is highly conserved across species, indicating its crucial role in maintaining neurofibromin’s structural integrity and function [34]. Together, these computational methods provided strong evidence for a genotype–phenotype correlation, helping to explain the severity and early onset of CPT observed in the affected family members. These insights also hold significant clinical implications, highlighting the potential of early genetic screening to identify at-risk individuals and the future possibility of targeting *NF1* loss-of-function mutations through precision gene therapy [47].

Despite these advancements, the study has notable limitations. The mutation was confirmed using blood samples, but lesion or periosteal tissue was not analyzed—potentially overlooking somatic mosaicism. Furthermore, while WES is a robust tool, it may miss non-coding or intronic variants that could also influence gene expression and disease development. The focus on a single family limits the ability to generalize the findings, and no functional studies (e.g., in vitro or in vivo models) were conducted to validate the biological effects of the mutation. The variability in symptoms among family members also points to possible modifier genes or environmental factors, which were not investigated [47].

In conclusion, these studies demonstrate how AI-powered and standard bioinformatic tools can significantly advance our understanding of *NF1* gene mutations and their role in rare disorders like CPT. By integrating mutation detection, structural modeling, and conservation analysis, they not only clarified the mutation’s impact but also enhanced diagnostic precision and opened avenues for personalized treatment strategies in the future.

Table 1 below provides a comprehensive overview of the current AI tools reviewed in this article for *NF1* variant analysis. These tools are categorized based on their primary function: Variant Interpretation and Pathogenicity Prediction, Protein Structure and Stability Prediction, Tumor Classification, and Therapeutic Prediction.

## 5. Future Directions

### 5.1. NGS for Genotype–Phenotype Correlation

In NF1, understanding how specific genetic mutations relate to clinical features is essential for improving diagnosis, risk assessment, and individualized treatment. Despite over 3197 pathogenic *NF1* variants being identified, only about 10–15% of cases show clear genotype–phenotype correlations [48]. These correlations, however, offer meaningful clinical insights. For example, individuals with the *p.Met992del* variant usually exhibit mild symptoms such as CALMs and axillary freckling, but typically lack cutaneous or plexiform neurofibromas, making them unsuitable candidates for neurofibroma-targeted clinical trials [48,49]. Similarly, mutations like *p.Arg1809Cys* are associated with pigmentary changes and Noonan-like features without tumor development [48,50]. In contrast, patients with large *NF1* microdeletions, especially the type 1 deletion spanning 1.4 Mb and including genes like *SUZ12* and *R*NF1*35*, tend to experience more severe disease involving numerous tumors, cognitive impairment, overgrowth, cardiovascular anomalies, and a fourfold increased risk of MPNST [48,51]. Germline mosaicism also influences disease severity, with mosaic individuals often presenting milder features than non-mosaic carriers of the same variant [52]. Additionally, missense mutations in codons 844–848 of the CSRD correlate with severe phenotypes including optic gliomas, plexiform neurofibromas, and skeletal abnormalities, underscoring that pathogenicity is not limited to classic functional domains [48,53]. Frameshift mutations were notably associated with cognitive impairment, while nonsense mutations often coincided with skeletal deformities. Whole-gene deletions and duplications were further linked to spinal abnormalities, cardiovascular complications and a higher likelihood of multiple systemic tumors [54].

Regardless of these associations, NF1 remains highly variable in presentation, even among patients with the same mutation, due to modifying factors such as somatic second hits, epigenetic alterations, and environmental influences. Malignancies like MPNST often develop through a multistep genetic process involving the somatic loss of genes like *NF1*, *CDKN2A/B*, *SUZ12*, or *EED*, combined with epigenetic deregulation, including the loss of *H3K27me3* and hypermethylation of key genes like *SOX10* and *CDKN2A* [48]. Given the intricate genetic and epigenetic landscape of *NF1*, advanced and scalable technologies are crucial for thoroughly characterizing its diverse mutational spectrum and associated phenotypic variability.

As previously discussed, NGS has revolutionized genomic research [10,11,13]. However, despite its advanced capabilities, NGS still faces significant challenges, particularly in the interpretation of VUS and in translating genomic findings into clinically actionable insights. To help address these limitations, AI is increasingly being integrated into genomic workflows, enhancing the accuracy, scalability, and efficiency of NGS data analysis across several key processes, including variant calling, annotation, pathogenicity prediction, and the linking of genotype to phenotype [27].

Traditionally, bioinformatics tools, used to process and analyze NGS data, have relied on rule-based algorithms and statistical models. In contrast, machine learning and deep learning approaches introduce data-driven models capable of learning complex patterns directly from the data itself. This allows AI systems to perform tasks with greater adaptability and scalability, which is especially valuable when working with large, noisy, or incomplete datasets such as those encountered in *NF1* research. The integration of AI into NGS workflows was comprehensively reviewed last year by Choon et al. [27], who highlighted a range of AI-based tools that have demonstrated promising results across various stages of the genomic analysis pipeline [27].

Firstly, variant calling, the process of detecting genetic variants from sequencing data, has traditionally relied on algorithms using fixed heuristic rules. AI models, however, can learn directly from sequencing outputs, thereby improving accuracy and reducing false discovery rates. For example, DeepVariant [55] employs convolutional neural networks (CNNs) to transform aligned sequencing reads into image representations, leveraging computer vision techniques for variant identification. Similarly, Clairvoyante [56], another CNN-based model, extends these capabilities to long-read sequencing technologies, enhancing variant detection in complex genomic regions. DeepNano [57] utilizes recurrent neural networks (RNNs) to improve base calling accuracy in nanopore sequencing, while NeoMutate [58] applies ensemble learning with Bayesian classifiers and other supervised ML algorithms to optimize variant detection through the integration of diverse sequence features.

Secondly, in the variant filtering stage, AI tools outperform traditional methods by learning from labeled datasets to distinguish true variants from sequencing artifacts. SNooPer [59], which uses random forest algorithms, is specifically designed for detecting somatic variants in low-coverage data. GARFIELD-NGS [60] applies machine learning to effectively separate true variants from false positives in exome sequencing data. Meanwhile, Intelli-NGS [61], powered by deep neural networks, further refines this process by maintaining high sensitivity while minimizing false positives and negatives, thereby increasing confidence in the final variant set.

Thirdly, variant annotation and prioritization, critical steps for assessing the clinical relevance of detected variants, also benefit significantly from AI-based approaches. Whereas traditional annotation tools rely on manually curated databases and fixed scoring systems, AI models can learn complex relationships between variant features and pathogenic outcomes. For instance, Skyhawk [56], a deep neural network model, simulates expert variant review processes to prioritize clinically actionable variants. DANN [62] applies deep learning to predict the pathogenic potential of genetic variants, outperforming traditional classifiers like support vector machines, and DeepSEA [63] extends these capabilities to non-coding regions, using CNNs to predict the regulatory impact of non-coding variants directly from sequence context.

Additionally, one of the key strengths of AI-driven approaches is their ability to incorporate phenotype information into variant prioritization, which improves the interpretation of sequencing results within a clinical context. For example, DeepGestalt [64], a CNN-based model, uses facial phenotypic analysis to assist in diagnosing over 200 genetic syndromes, demonstrating the power of integrating phenotypic data with genomic analysis. Similarly, DeepPVP [65] combines phenotypic and genomic data through deep neural networks for enhanced variant prioritization, while Xrare [66] employs machine learning models to jointly analyze phenotype–genotype associations for identifying pathogenic variants in rare disorders.

The fundamental distinction between traditional bioinformatics and AI-based methods lies not only in their methodologies but also in their flexibility and learning capacity. Conventional bioinformatics tools often require manual parameter tuning and rely on predefined rules. In contrast, AI models can adaptively learn from data, continuously improving as more annotated datasets become available. This adaptability makes AI-driven approaches particularly well suited for addressing the complexities of genomic data, especially in the context of diseases like NF1, where data scarcity and high genetic heterogeneity often limit the effectiveness of traditional analysis strategies [27].

Moreover, AI facilitates the integration of genomic data with electronic health records, supporting precision medicine and personalized care strategies. Its advantages include the automation of complex tasks, reduction in human error in variant interpretation, and the discovery of novel correlations between genetic mutations and clinical outcomes. Nonetheless, AI has limitations—it requires large, annotated datasets for training, demands high computational resources, and presents ethical and privacy challenges in clinical implementation [27].

**Figure 1:**
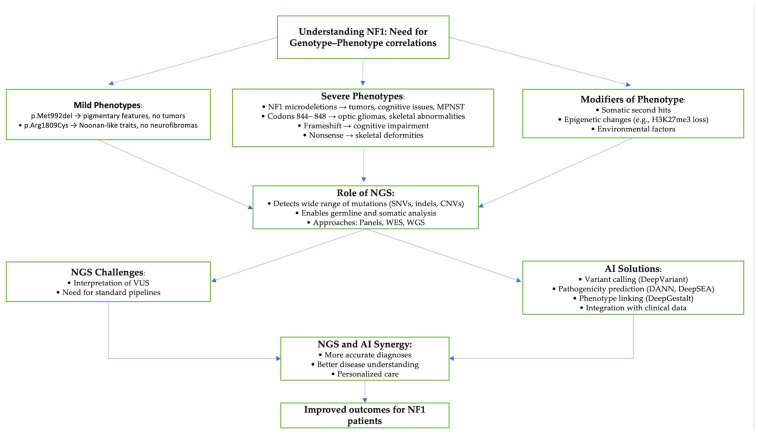
Genotype—phenotype correlation in NF1 and role of NGS and AI in precision medicine.

The synergy between NGS and AI, as seen well in Figure 1 below, forms a powerful foundation for advancing genotype–phenotype correlations and genomic medicine. While NGS generates massive amounts of genetic data, AI extracts clinically meaningful insights, enabling more accurate diagnoses, better disease understanding, and tailored treatment plans. In NF1, this integrated approach is expanding our ability to define genotype–phenotype relationships and refine therapeutic strategies. Although only a subset of *NF1* variants currently have established clinical correlations, continued advances in genomic technologies and AI promise to deepen our understanding and improve outcomes for patients with NF1 and other rare genetic conditions [27].

### 5.2. Multi-Omics

Recent advances in genomics, AI, and precision medicine have paved the way for innovative strategies with strong potential to improve the understanding and management of NF1 in the future. Although these technologies have not yet been directly applied to NF1, they represent important methodological breakthroughs that could conceptually enhance variant classification, tumor stratification, and personalized care for this disorder.

A notable example of such progress is the integrative multi-omics study by Yang et al. [67], which conducted CNV, DNA methylation, and microRNA (miRNA) expression profiling to classify tumor subtypes and identify immune-related biomarkers in lower-grade glioma. In this study, MOVICS (Multi-Omics Integration and Visualization in Cancer Subtyping) was employed as the integrative clustering method to merge genome-wide profiling data from these three omics layers. Using this approach, the authors identified four distinct molecular subtypes of lower-grade glioma, each significantly associated with patient prognosis, immune-related features, and genetic characteristics. By integrating multi-omics data, the study provided a robust classification framework and highlighted the critical role of miRNA dysregulation driven by genomic and epigenomic alterations. This work demonstrates how the integration of multiple layers of genomic and epigenomic information can reveal key regulatory mechanisms and prognostic markers, ultimately supporting the development of personalized treatment strategies. Given the tumor heterogeneity and diverse clinical manifestations observed in NF1, adopting similar multi-omics approaches could greatly enhance patient stratification, facilitate the discovery of prognostic biomarkers, and improve our understanding of immune evasion mechanisms in NF1-associated tumors.

Further expanding the possibilities for precision variant interpretation, the sc2GWAS platform introduced by Yin et al. [68] integrates genome-wide association study data with single-cell RNA sequencing to achieve cell-specific mapping of genetic risk variants. This enables the identification of precise trait–cell–gene relationships and highlights the functional impact of variants within specific cellular contexts. Applying this strategy to NF1 could facilitate the discovery of disease-relevant variants within key cell types, such as Schwann cells or neural crest-derived populations implicated in tumorigenesis and neurodevelopment.

In the field of precision modeling and real-time symptom assessment, Xing et al. [69] demonstrated the use of AI-based sensors, wearable devices, and machine learning algorithms for the dynamic monitoring and adjustment of pain management strategies. These technologies allow for continuous data collection and individualized treatment planning based on real-time physiological feedback. Such an approach could be adapted to support symptom monitoring in NF1 patients, particularly for managing chronic pain, nerve dysfunction, or tracking treatment responses, thereby enhancing individualized patient care.

Complementary to these AI-based approaches, recent research has also provided insights into cellular function, stress responses, and epigenetic regulation that may inform future NF1 studies. The work by Zhou et al. [70] highlights the role of *Nynrin* in maintaining hematopoietic stem cell (HSC) function through the regulation of mitochondrial permeability transition pore opening, a mechanism relevant to cell survival under stress conditions. In this study, the authors used RNA-seq, single-cell RNA-seq (scRNA-seq), and ChIP-seq as key omics tools to uncover the role of *Nynrin* in HSC function. These approaches allowed for the identification of *Nynrin* target genes, and revealed critical pathways involved in mitochondrial regulation and stem cell maintenance. The use of multi-omics was essential to provide a comprehensive and high-resolution understanding of gene regulation and cellular mechanisms, highlighting the power of integrative omics in uncovering complex biological processes. Additionally, Zhou et al. [71] investigated the impact of METTL3-modified exosomes and m6A RNA methylation on cellular proliferation and migration, providing further understanding of how post-transcriptional modifications can influence tumor biology. Although these studies do not directly involve NF1, their focus on cellular stress mechanisms and epigenetic regulation may offer valuable perspectives for understanding NF1-related tumorigenesis and developing novel therapeutic approaches.

Together, these advancements in AI-integrated multi-omics analysis, single-cell variant mapping, precision modeling, and epigenetic research offer a conceptual framework that could significantly contribute to NF1 research in the future. Their potential to improve variant discovery, patient stratification, symptom monitoring, and targeted therapy development highlights their relevance for advancing personalized medicine in this complex genetic disorder.

## 6. Bias of AI in Genetics

AI models have become powerful tools for genetic variant interpretation, offering the potential to improve diagnostic accuracy and support clinical decision-making. However, these models are vulnerable to systemic biases when trained on non-diverse datasets, limiting their generalizability across different ethnic groups. This issue is particularly critical in the context of NF1, a disorder with a highly heterogeneous mutation spectrum, including many rare, de novo, and population-specific variants.

Several widely used AI-based tools for variant classification, such as VEST3, REVEL, and ClinPred, have demonstrated strong performance for missense variants, yet as shown by Accetturo et al. [29], their predictive accuracy is influenced by the composition of their training data—largely sourced from populations of European ancestry. This lack of diversity compromises the ability of these models to correctly classify variants that are more prevalent or unique to underrepresented groups, leading to potential misinterpretation and diagnostic uncertainty [29].

A key contributor to this problem is the dependence of AI models on public variant databases, such as ClinVar and the HGMD, for training and validation. While these databases are essential resources for variant annotation, studies have shown that they contain ancestry-related biases. Work by Sharo et al. [72] revealed that both ClinVar and HGMD include pathogenic classifications that do not always reflect real-world disease prevalence, particularly in individuals of African ancestry, who were disproportionately flagged as affected by rare disorders such as inborn errors of metabolism (IEMs). This misclassification stems from the underrepresentation of diverse populations during variant curation and a historical reliance on European-derived data. Although updated allele frequency guidelines have reduced some of these errors, common benign variants in African genomes remain at risk of being mislabeled as pathogenic, especially in HGMD where reclassification rates are slower compared to ClinVar [72].

Since many AI models rely on public variant databases as ground truth, misclassifications within these databases can be propagated and amplified by predictive algorithms, further compromising model reliability, especially for individuals from non-European populations. This feedback loop not only reduces the accuracy of AI-driven variant interpretation but also risks reinforcing existing health disparities in genomic medicine.

Efforts to address these biases have emphasized the importance of both functional validation and equitable data representation. For example, the study by Dawood et al. [73] found that individuals of non-European ancestry carry a significantly higher burden of VUS, largely due to underrepresentation in genomic databases. Using the experimental platform known as MAVEs (Multiplexed Assays of Variant Effects), the authors were able to reclassify a greater proportion of VUS in non-European individuals than in their European counterparts, thereby reducing diagnostic uncertainty and helping to mitigate data imbalance. However, the study also highlighted that while MAVE-generated functional data were applied equitably across ancestries, other evidence sources, such as allele frequency data and computational predictions, continued to perform inequitably, with a bias toward European genetic backgrounds [73].

Within this framework, community-driven data-sharing platforms like Franklin by Genoox [74] represent a valuable strategy to mitigate bias in AI-based variant interpretation by enhancing the diversity and accuracy of the underlying evidence used for model training and validation. The study by Einhorn et al. [75] exemplifies how leveraging the Franklin platform, which integrates large-scale real-world sequencing data with community-contributed variant classifications, enabled the identification of novel pathogenic founder variants (PFVs) absent from traditional carrier screening panels and global resources like ClinVar. By combining automated ancestry inference with evidence shared across a broad network of clinical users, the platform facilitates the detection of true pathogenic variants within underrepresented groups while also supporting the reclassification of variants that may have been misinterpreted due to limited or biased data. By incorporating variant observations from diverse ancestries and linking them to clinical phenotypes, it helps prevent the entrenchment of misclassified or uncertain variants within AI training datasets—a key contributor to systemic bias in predictive models. This participatory framework not only enhances the accuracy of pathogenicity assessments but also reduces the burden of VUS in non-European populations, where data gaps are most pronounced. This approach directly addresses the well-documented limitations of static variant databases by allowing continuous updates and real-world feedback, significantly improving the interpretive landscape for AI tools reliant on these resources [75].

## 7. Strengths and Limitations

Table 2 and Table 3 below provide an overview of the advantages and drawbacks of utilizing AI in the context of *NF1* analysis, compiled by the authors based on the previously discussed data to highlight both the potential benefits, such as enhanced diagnostic accuracy and efficiency, and the current challenges, including data quality, interpretability, and clinical integration.

## 8. AI Diagnosing NF1 Beyond Genetics

While the following is not a genetics-focused study, it is essential to highlight the growing role of AI in enhancing NF1 diagnostics in a holistic approach. A groundbreaking multicenter study published in 2025 introduced deep learning models trained on T2-weighted MRI data to distinguish between benign plexiform neurofibromas (PNFs) and MPNSTs, a clinically critical differentiation. The researchers analyzed over 3100 MRI images from 347 patients across seven medical centers in China, establishing this as the largest image-based AI study in NF1 to date. Recognizing that NF1 tumors can arise throughout the body and exhibit highly variable imaging features, the team developed a novel one-step AI model that integrates both tumor detection and classification, while incorporating contextual information from surrounding normal tissues to improve diagnostic precision [76].

The study employed a traditional two-step deep learning imaging framework using U-Net for lesion segmentation and ResNet18 for classification. However, the real innovation was the introduction of a streamlined, YOLO-v5-based one-step model, which completed segmentation and diagnosis simultaneously. This model achieved impressive results—an 85.71% accuracy in the validation set and 84.75% in an independent test set—while requiring only a third of the computational resources of previous models. Its ability to mimic clinician reasoning by factoring in both anatomical location and lesion context allowed for enhanced reliability, even in complex regions such as the head and face. The use of Grad-CAM interpretability tools further validated that the AI focused on relevant tumor features, aligning its “attention” with clinical expectations [76].

This study reflects a meaningful evolution in NF1 diagnostics, highlighting a shift from a traditional focus on genetics and clinical symptoms toward the integration of AI-powered imaging tools. While genetic testing remains a key component in identifying NF1 variants, its limitations—particularly the lack of consistent genotype–phenotype correlations—accentuate the need for additional diagnostic strategies. AI is now becoming an integral part of the multidisciplinary approach to NF1, expanding beyond genetics to include advanced imaging analysis as a critical layer of insight. This progression points toward a future where AI plays a leading role in enhancing diagnostic precision, enabling earlier detection, and supporting more personalized care for individuals with this highly variable condition.

## 9. Discussion

NF1 continues to pose significant diagnostic and therapeutic challenges due to its marked genetic heterogeneity, variable phenotypic expression, and the intrinsic complexity of the *NF1* gene itself [17]. While the NIH diagnostic criteria remain clinically valuable, they are often insufficient in cases with early onset, atypical manifestations, or borderline presentations [6]. In such instances, molecular testing has become increasingly indispensable. However, the interpretation of *NF1* variants remains difficult since the gene lacks well defined mutation hotspots and encompasses thousands of unique variants, many of which are classified as VUS [31]. This high rate of uncertain findings complicates clinical decision-making and diminishes the immediate utility of genetic test results.

Recent advancements in AI and computational biology are transforming the landscape of NF1 diagnostics, particularly in the interpretation of gene variants. AI-driven tools such as VEST3, REVEL, ClinPred, DITTO, and RENOVO-NF1 have demonstrated high accuracy in classifying genetic variants and reclassifying VUS with improved reliability [29,37,41]. These models leverage diverse inputs—including structural, functional, and evolutionary data—to generate more refined pathogenicity predictions. Further, AI’s role extends beyond simple classification. In silico modeling platforms like AlphaFold allow researchers to predict the biophysical impact of mutations on neurofibromin, as demonstrated in the identification of destabilizing mutations such as *C379R* and *C1016Y*, that might otherwise remain undetected or misclassified by conventional methods [31]. Such tools offer a promising path forward in resolving diagnostic ambiguity and expanding the catalog of clinically actionable variants.

In addition to variant interpretation, AI has shown potential in addressing other clinically significant aspects of NF1 management. For example, LTC-based machine learning models have achieved near-perfect accuracy in classifying NF1-associated tumors using gene expression data [39]. The RENOVO-NF1 tool has also made a substantial impact by reclassifying more than 4000 missense VUS listed in ClinVar, demonstrating its utility in refining variant databases and enhancing clinical interpretation [41]. In rare and complex manifestations such as CPT, AI-powered in silico trials and bioinformatics platforms have been used to predict treatment response and identify potential therapeutic targets, thereby contributing to emerging precision medicine strategies in NF1 [46].

Furthermore, AI is increasingly being utilized beyond genomics for NF1, extending into the field of medical imaging. Deep learning models can accurately distinguish malignant from benign lesions on whole-body MRI scans in patients with NF1 [76]. These models, trained to account for complex anatomical backgrounds, underline the expanding role of AI across disciplines and highlight its capacity to complement both genetic and radiologic diagnostics. The integration of AI across these domains reinforces the multidisciplinary nature of NF1 care and reflects the broader goal of enhancing diagnostic accuracy and patient outcomes.

The advantages of applying AI to NF1 are numerous (Table 2). AI algorithms can process vast and complex datasets with remarkable speed, significantly reducing the time and labor associated with manual variant interpretation. They are capable of detecting subtle patterns and correlations that may be missed by human analysis, offering insights into potential genotype–phenotype relationships. With continued development, AI could enable the integration of genomic, transcriptomic, and phenotypic data, helping clinicians build comprehensive and individualized patient profiles that support personalized care.

Nonetheless, several limitations and challenges remain (Table 3). Many AI models are trained on datasets that lack sufficient population diversity, potentially limiting their generalizability across ethnic and demographic groups. Moreover, while these tools show strong predictive performance, most predictions lack functional validation and, therefore, cannot yet be translated directly into clinical recommendations. This is especially true for non-coding and intronic variants, which are currently underrepresented in algorithm training and interpretation. AI systems also require high-quality, annotated datasets for effective training, and their successful implementation into clinical workflows will depend on overcoming regulatory, infrastructural, and ethical hurdles. In this evolving landscape, platforms like Franklin by Genoox [74], which offer real-time variant classification and community-based data sharing, represent valuable tools for enhancing collaborative interpretation and reducing uncertainty in variant analysis. Moreover, concerns about data privacy, algorithm transparency, and clinician familiarity with proper oversight of AI tools is crucial to guarantee their safe and efficient integration into healthcare settings [27].

Although AI is not yet widely implemented in routine clinical practice for establishing genotype–phenotype correlations in NF1, it holds significant promise in transforming the field [47]. As the interpretation of *NF1* gene variants continues to be complicated by the gene’s size, the diversity of mutations, and the clinical variability of the disorder, AI-powered tools offer the potential to streamline variant classification, prioritize pathogenic mutations, and support early diagnostic efforts [27]. Emerging in silico models and machine learning algorithms have already shown encouraging results in reclassifying VUS and identifying patterns within genomic and transcriptomic data that may eventually correlate with clinical features.

Despite these challenges, the growing application of AI in both genetic and imaging domains of NF1 reflects the clinical urgency and importance of improving diagnostic precision and therapeutic outcomes. These multidisciplinary efforts—from molecular variant analysis to advanced imaging interpretation—are collectively advancing the field toward more personalized, data-driven care. Ultimately, the integration of AI into NF1 diagnostics holds great promise for enhancing the quality of life and long-term prognosis of individuals living with this complex genetic disorder.

## 10. Conclusions

While these advances remain largely at the research stage, they highlight a future in which AI could complement NGS technologies to improve diagnostic precision and support individualized treatment approaches for NF1. Ongoing efforts in the development, validation, and incorporation of AI tools into genetics and clinical practice will be vital to fully harness their potential in the diagnosis and management of NF1.

## Figures and Tables

**Table 1:** AI tools used in identifying *NF1* variants based on their primary function.

**Variant Interpretation and Pathogenicity Prediction**	
**AI Tool**	**Function/Description**
SpliceAI [22]	Deep learning model that predicts splice site disruptions across the entire transcript, enabling accurate detection of both canonical and non-canonical splice-altering variants in the *NF1* gene.
REVEL [19,29]	Ensemble machine learning tool that combines scores from multiple individual predictors (e.g., SIFT, PolyPhen-2) to improve the classification of rare missense variants as likely benign or pathogenic.
VEST3 [29,30]	Supervised learning algorithm trained on known pathogenic and benign variants; uses sequence conservation, protein features, and structural data to predict functional impact of *NF1* missense mutations.
ClinPred [28,29]	Machine learning classifier trained on ClinVar data; integrates multiple features including conservation, protein annotations, and clinical evidence to assess variant pathogenicity.
PredictSNP2 [31]	Consensus-based predictor that merges results from several established tools (e.g., SNAP, PANTHER, PhD-SNP) to enhance reliability in predicting the functional consequences of *NF1* missense variants.
Align-GVGD [31]	Combines evolutionary conservation and biochemical properties to assess the functional impact of amino acid substitutions in *NF1*, particularly useful in cysteine mutation evaluation.
RENOVO-NF1 [41]	*NF1*-specific random forest model that calculates a Pathogenicity Likelihood Score (PLS) and effectively reclassifies *NF1* missense VUS into likely pathogenic or benign with high accuracy.
DITTO [37]	Advanced AI model that integrates transcriptomic, proteomic, and structural dynamics data to evaluate the functional effects of *NF1* mutations, including protein conformation-specific impacts.
SAAFEC-seq [37]	Gradient boosting-based model estimating protein stability changes (ΔΔG) using sequence-derived features to assess potential pathogenic effects of *NF1* mutations.
**Protein Structure and Stability Prediction**	
**AI Tool**	**Function/Description**
AlphaFold3 [31,35]	Deep learning tool for predicting 3D protein structures at high resolution; used to visualize and assess how *NF1* mutations affect neurofibromin folding and domain architecture.
iStable [31]	Integrates predictions from iMutant 2.0 and MUpro to estimate mutation-induced changes in protein stability (ΔΔG), helping identify destabilizing *NF1* variants.
iMutant 2.0 [31]	SVM-based predictor for estimating the impact of single-point mutations on protein stability.
MUpro [31]	Combines SVM and neural networks to predict whether a mutation increases or decreases protein stability in *NF1*.
**Tumor Classification**	
**AI Tool**	**Function/Description**
GENIE-NF-AI [39]	Deep learning model based on a liquid neural network (LSTM) trained on gene expression data to classify NF1-associated tumors with high accuracy. It integrates black-box predictive performance with glass-box interpretability—using explainable AI layers to clarify how gene features contribute to classification, thus enhancing clinical trust and transparency.
**Therapeutic Prediction**	
**AI Tool**	**Function/Description**
In Silico AI Tools [46]	Machine learning framework used in virtual clinical trials for NF1-related CPT; includes random forest for response prediction, biomarker discovery, and patient stratification based on simulated biological outcomes.

**Table 2:** Advantages of AI in *NF1* gene analysis.

AI Advantage	Description	Example/Application in NF1
**Enhanced Variant Interpretation**	AI reduces uncertainty in classifying missense mutations and VUS.	Tools like REVEL, VEST3, and RENOVO-NF1 improve confidence in variant classification, aiding early diagnosis and risk assessment [29,41].
**Accurate Structural Impact Prediction**	AI-powered structural models predict how mutations affect neurofibromin conformation.	AlphaFold3 and DITTO reveal stability changes in different protein states, offering insights for targeted therapies [31,35,37].
**Rapid Analysis of Big Genomic Data**	AI accelerates processing of sequencing datasets, prioritizing clinically relevant variants.	In silico tools rapidly stratify patient data (e.g., CPT models in virtual trials), reducing diagnostic delays [46].
**Integration of Multi-Omics Data**	AI can unify genomic, transcriptomic, and proteomic information for comprehensive variant assessment.	DITTO integrates transcriptomic and structural dynamics to model protein behavior across conformations [37].
**Support for Clinical Decision-Making**	AI enhances diagnostic precision and treatment planning by reducing ambiguity.	GENIE-NF-AI and RENOVO-NF1 assist in tumor classification and VUS reclassification, guiding early interventions [39,41].
**Ethical Therapeutic Exploration**	AI enables virtual clinical trials in populations where real trials are ethically challenging.	In silico BMP therapy trials for NF1-CPT model treatment outcomes in children without physical risk [46].

**Table 3:** Challenges and mitigation strategies for AI in *NF1* analysis.

Challenge	AI Limitation	Proposed Mitigation Strategy
**Data Representation Bias**	AI tools trained on databases like ClinVar and HGMD often reflect Eurocentric variant data, reducing performance on variants common in non-European populations [72].	Promote use of diverse datasets and platforms like Franklin; integrate community-contributed variant data for broader ancestry coverage [74].
**Limited Generalizability**	AI models may fail on novel or ultra-rare variants due to lack of similar examples in training data [72].	Continually retrain models with updated real-world clinical data and include synthetic data from simulated environments where appropriate [74].
**Lack of Functional Validation**	AI predictions often lack biological validation, reducing clinical trust.	Use multiplexed assays of variant effects (MAVEs) and encourage AI–wet lab partnerships to validate predictions [73].
**Missense Variant Focus**	Most tools are optimized for missense mutations and lack support for intronic, splicing, or structural variants.	Incorporate tools like SpliceAI to cover splicing and regulatory regions [22].
**Interpretability and Clinical Trust**	Black-box models limit clinical adoption due to poor transparency in decision-making [39].	Use explainable AI (e.g., GENIE-NF-AI’s glass-box overlay) to make model logic transparent for clinicians [39].
**Regulatory and Integration Barriers**	Many AI tools are not validated for clinical use, delaying integration into routine diagnostics [39,41].	Develop standards for AI validation and interoperability in genomics workflows, aligned with ACMG frameworks.

## Abbreviations

Neurofibromatosis Type 1 (NF1), artificial intelligence (AI), National Institutes of Health (NIH), attention-deficit/hyperactivity disorder (ADHD), unidentified bright objects (UBOS), malignant peripheral nerve sheath tumors (MPNSTs), gastrointestinal stromal tumors (GISTs), café-au-lait macules (CALMs), next-generation sequencing (NGS), neurodevelopmental disorders (NDDs), variants of uncertain significance (VUS), disease-causing genes (DCGs), cysteine-serine-rich domain (CSRD), stability changes (ΔΔG), Pleckstrin Domain (PH), American College Of Medical Genetics And Genomics (ACMG), Deep Integration For Transcriptomic And Translational Omics (DITTO), Single-Nucleotide Variants (SNVs), liquid neural network (LTC), congenital pseudarthrosis of the tibia (CPT), bone morphogenetic protein (BMP), whole-exome sequencing (WES), whole-genome sequencing (WGS), convolutional neural networks (CNNs).
